# Performance evaluation of fifth-generation ultra-high-resolution SPECT system with two stationary detectors and multi-pinhole imaging

**DOI:** 10.1186/s40658-020-00335-6

**Published:** 2020-11-02

**Authors:** Jan V. Hoffmann, Jan P. Janssen, Takayuki Kanno, Takayuki Shibutani, Masahisa Onoguchi, Constantin Lapa, Jan-Peter Grunz, Andreas K. Buck, Takahiro Higuchi

**Affiliations:** 1 Department of Nuclear Medicine, University Hospital Würzburg, Oberdürrbacher Strasse 6, 97080 Würzburg, Germany; 2 Comprehensive Heart Failure Center, University Hospital Würzburg, Würzburg, Germany; 3 Department of Quantum Medical Technology, Graduate School of Medical Sciences, Kanazawa University, Kanazawa, Japan; 4 Nuclear Medicine, Medical Faculty, University of Augsburg, Augsburg, Germany; 5 Department of Diagnostic and Interventional Radiology, University Hospital Würzburg, Würzburg, Germany; 6 Graduate School of Medicine, Dentistry and Pharmaceutical Sciences, Okayama University, Okayama, Japan

**Keywords:** Small-animal imaging, SPECT, Mouse, Collimator, Post-reconstruction filtering

## Abstract

**Background:**

Small-animal single-photon emission computed tomography (SPECT) systems with multi-pinhole collimation and large stationary detectors have advantages compared to systems with moving small detectors. These systems benefit from less labour-intensive maintenance and quality control as fewer prone parts are moving, higher accuracy for focused scans and maintaining high resolution with increased sensitivity due to focused pinholes on the field of view. This study aims to investigate the performance of a novel ultra-high-resolution scanner with two-detector configuration (U-SPECT5-E) and to compare its image quality to a conventional micro-SPECT system with three stationary detectors (U-SPECT^+^).

**Methods:**

The new U-SPECT5-E with two stationary detectors was used for acquiring data with ^99m^Tc-filled point source, hot-rod and uniformity phantoms to analyse sensitivity, spatial resolution, uniformity and contrast-to-noise ratio (CNR). Three dedicated multi-pinhole mouse collimators with 75 pinholes each and 0.25-, 0.60- and 1.00-mm pinholes for extra ultra-high resolution (XUHR-M), general-purpose (GP-M) and ultra-high sensitivity (UHS-M) imaging were examined. For CNR analysis, four different activity ranges representing low- and high-count settings were investigated for all three collimators. The experiments for the performance assessment were repeated with the same GP-M collimator in the three-detector U-SPECT^+^ for comparison.

**Results:**

Peak sensitivity was 237 cps/MBq (XUHR-M), 847 cps/MBq (GP-M), 2054 cps/MBq (UHS-M) for U-SPECT5-E and 1710 cps/MBq (GP-M) for U-SPECT^+^. In the visually analysed sections of the reconstructed mini Derenzo phantoms, rods as small as 0.35 mm (XUHR-M), 0.50 mm (GP-M) for the two-detector as well as the three-detector SPECT and 0.75 mm (UHS-M) were resolved. Uniformity for maximum resolution recorded 40.7% (XUHR-M), 29.1% (GP-M, U-SPECT5-E), 16.3% (GP-M, U-SPECT^+^) and 23.0% (UHS-M), respectively. UHS-M reached highest CNR values for low-count images; for rods smaller than 0.45 mm, acceptable CNR was only achieved by XUHR-M. GP-M was superior for imaging rods sized from 0.60 to 1.50 mm for intermediate activity concentrations. U-SPECT5-E and U-SPECT^+^ both provided comparable CNR.

**Conclusions:**

While uniformity and sensitivity are negatively affected by the absence of a third detector, the investigated U-SPECT5-E system with two stationary detectors delivers excellent spatial resolution and CNR comparable to the performance of an established three-detector-setup.

## Introduction

Compared to positron emission tomography (PET), the main advantage of micro single-photon emission computed tomography (micro-SPECT) becomes apparent in ultra-high-resolution submillimetre imaging. However, the benefit of superior resolution traditionally comes at the cost of losing sensitivity [1–3]. This phenomenon is called resolution-sensitivity trade-off and limits in particular the use of micro-SPECT in preclinical imaging with small rodents [4].

In recent years, the progression of multi-pinhole collimated SPECT systems enabled imaging of animals with high spatial resolution while keeping the deficit in sensitivity to a reasonable degree. This was essential for the application of SPECT to numerous well-established disease models in small rodents. With improved sensitivity, increasing the tracer injection dose to maintain enough signal was no longer necessary [3, 5].

In addition to multi-pinhole collimation, the development of ultra-high-resolution SPECT (U-SPECT) scanners is lately focused on stationary instead of moving detectors, usually arranged in a triangle configuration to cover 360° of detection area. The goal of this set-up is to reduce the amount of moving parts for higher precision and less elaborate calibration and maintenance. Moreover, this allows the region of interest to be measured very precisely, which helps with focused imaging [5–10]. In contrast to conventional systems operating three detectors, the novel fifth-generation U-SPECT system tested in this study uses a two-detector set-up without the bottom detector. The main reason for removing the third bottom detector is to reduce acquisition and material costs since it is assumed that the detector’s efficiency is sufficiently high.

The aim of this study was to analyse whether the two-detector U-SPECT system could provide the same ultra-high-resolution for submillimetre molecular imaging in small rodents as a conventional three-detector micro-SPECT-system, despite considerably less detection surface.

## Materials and methods

### System description

In this study, a novel fifth-generation U-SPECT system (U-SPECT5/CT E-Class; MILabs, Utrecht, The Netherlands), in the following referred to as “2DT-Scanner” was investigated with focus on its SPECT performance. The scanner construction is similar to the previous generation micro-SPECT system (U-SPECT^+^; MILabs) [7] and consists of stationary detectors with an exchangeable multi-pinhole collimator. A XYZ-stage moving the scanned object in a spiral step-mode through the field of view (FOV) enables whole body and focused scans [6, 8, 9].

**Fig. 1:**
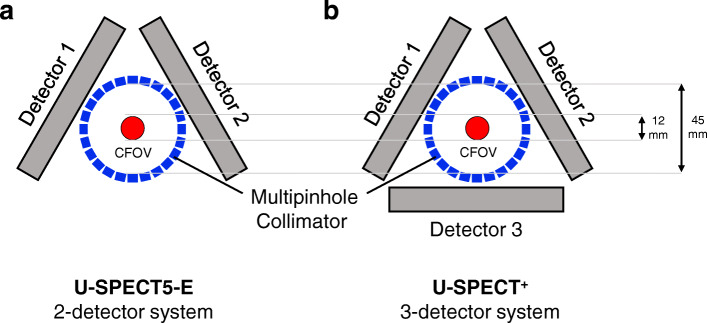
Schematic cross section of the arrangement of the detectors of the new two-detector SPECT system (2DT-Scanner) (**a**) and a standard three-detector system (3DT-Scanner) (**b**). The surface of each stationary detector head, shown here as a square, has a size of 47.2 cm × 59.5 cm and consists of 9.5-mm-thick NaI(Tl) crystals. Collimators’ inner diameter is 45.0 mm and centre field of view (CFOV) counts 12.0 mm diameter by 7.0 mm length

For the conventional U-SPECT5, the detectors are arranged as a triangle around the FOV. The detector surface comprises thallium-doped sodium iodide [NaI(Tl)] crystals and measures 47.2 cm by 59.5 cm with a 9.5 mm thickness each. The E-Class (U-SPECT5-E) differs from the normal version as the bottom detector is removed. This reduces the total detector surface by 1/3, from 8425.2 to 5616.8 cm^2^, what is still close to established micro-SPECT systems with stationary detectors [10]. Figure 1 shows the different detector arrangements schematically.

We investigated three different collimators for mice which are listed in Table 1. They all consist of tungsten with five rows of 15 pinholes, which focus on a central FOV of 7 mm axial length and 12 mm transaxial diameter [9]. The pinhole size of the collimators differs from 0.25 mm (type XUHR-M, MILabs), 0.60 mm (type GP-M, MILabs) to 1.00 mm (type UHS-M, MILabs).

**Table 1:** Specifications of the used collimators

Collimator type	XUHR-M	GP-M	UHS-M
Purpose	Extra ultra-high resolution mouse imaging	General purpose mouse imaging	Ultra-high sensitivity mouse imaging
Pinhole size (mm)	0.25	0.60	1.00
Number of pinholes	75		
Inner diameter (mm)	45		
Centre field of view—length (mm)	7		
Centre field of view—diameter (mm)	12		

All data was acquired in list-mode to enable selection of energy windows after acquisition. Quality control and maintenance was carried out on a regular basis as suggested by the manufacturer.

### Data processing and image reconstruction

For reconstruction of the created list-mode data, the similarity-regulated ordered-subsets expectation maximisation algorithm (SROSEM), as provided with the dedicated software (MILabs.Rec Version 8.06) of the 2DT-Scanner system, was used [11]. As no official recommendations were provided by the manufacturer, a validation of multiple reconstruction settings with respect to the image quality was carried out in advance. The number of iterations varied from 1 to 5, and the number of subsets remained constant at 128. As reference, images reconstructed with the same number of updates with the established maximum likelihood expectation maximisation (MLEM) algorithm with 1 subset and 128 to 640 iterations and pixel-based ordered-subset expectation maximisation (POSEM) algorithm with 32 subsets and 4 to 20 iterations were used [12, 13]. Image quality was compared both visually and quantitatively for noise and contrast using the methodology described below. Based on this, 3 iterations with 128 subsets were applied corresponding to the literature [11]. Smallest possible voxel size of 0.1 mm for XUHR-M and 0.2 mm for GP-M and UHS-M was used.

Photopeak window was set to 20%, resulting in a range of 126 to 154 keV for a photopeak of 140 keV. Scatter correction was performed by applying the triple energy window (TEW) methods [14]. The obtained SPECT images were analysed with the public domain program “A Medical Imaging Data Examiner” (AMIDE for Mac, version 1.0.5) [15], which was used for post-reconstruction 3D-Gaussian filtering as well as for placing and analysing regions of interest (ROIs).

### Sensitivity

The sensitivity of each collimator was calculated in accordance to the National Electrical Manufacturers Association (NEMA) [16] in photopeak counts per second (*R*_*i*_) per megabecquerel of applied activity (cps/MBq) by using a point source with an activity (*A*_*cal*_) of 1.9 ± 0.1 MBq (mean ± standard deviation) ^99m^Tc-pertechnetate was measured in a dose calibrator (ISOMED 2010, Nuvia Instruments, Dresden, Germany) with daily performed quality control and placed in the centre of the respective collimator [10]. All well counter measurements were decay corrected to the start of acquisition. Acquisition time was set to 5 min with 1 bed position (BP). All acquisitions had more than 100,000 counts to reduce statistical deviation.

$$ \mathrm{Sensitivity}=\frac{R_i}{A_{cal}} $$

### Spatial resolution

**Fig. 2:**
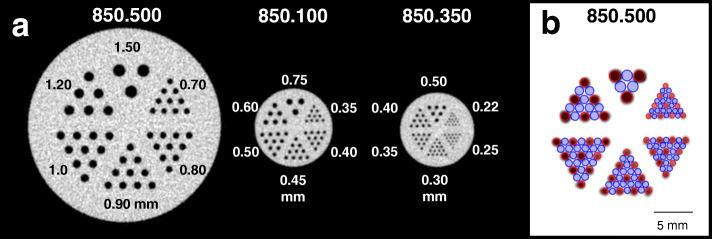
**a** Axial view of tomographic CT images of the three mini Derenzo hot-rod phantoms with different ranges of rod diameters, here shown in mm. All three phantoms were employed for spatial resolution assessment. Phantom 850.500 and 850.100 were also used to create templates for the contrast-to-noise ratio (CNR) analysis. The templates consisting of hot and cold regions of interest (ROI) were placed on the SPECT images of the respective phantoms. Each ROI had a length of 6.0 mm and a diameter of 90% of the corresponding rod diameter. **b** SPECT image plus CNR template derived from a 45-min scan of the phantom 850.500 acquired with the GP-M collimator and an activity concentration of 30 MBq/ml. The slice thickness was set to 6.0 mm

To investigate the collimator dependant system’s spatial resolution, three different mini Derenzo hot-rod phantoms, illustrated in Fig. 2, were scanned (Vanderwilt Techniques, Boxtel, Netherlands) (Table 2). Each phantom contains six different sized hot-rod capillary sections. The smallest phantom (850.350) covers rods with a diameter of 0.22 to 0.50 mm. The medium-sized phantom (850.100) includes rods with 0.35 to 0.75 mm and the biggest phantom (850.500) has the diameter range of 0.70 to 1.50 mm. In each section, the intercapillary distance equals the respective rod diameter of that section. The phantoms were filled with a ^99m^Tc-pertechnetate solution, activity concentration was 339.0 ± 39.1 MBq/ml, whereupon they were placed in the centre of the FOV. To cover the whole phantom, 9 BP for the 850.350 and 850.100 and 10 BP for the 850.500 phantom were needed with a total acquisition time of 45 min and 50 min, respectively. Smallest discriminable rod size was determined to describe the collimator-dependant maximum resolution.

**Table 2:** Specifications of the used phantoms

Phantom type	850.500	850.100	850.350
Purpose	Ultra-micro resolution phantom	Ultra-high micro resolution phantom	Ultra-high plus micro resolution phantom
Diameter of insert (mm)	24	12	12
Hight of insert (mm)	12	10	8
Diameter of hollow channels–range (mm)	0.22**–**0.50	0.35**–**0.75	0.70**–**1.50

### Uniformity

The system’s uniformity was assessed by using the phantoms mentioned above without the capillary inlays but filled with similar activity concentrations (322.8 ± 8.6 MBq/ml) of a ^99m^Tc-pertechnetate solution. The filling volume was 1.3 ml. Uniformity analysis was performed by positioning a ROI with a diameter of 7.5 mm and 6.0 mm length in the centre of the phantom. The image was smoothed by a Gaussian post-filter with full width at half maximum (FWHM) of 0.35 mm (XUHR-M), 0.50 mm (GP-M) and 0.75 mm (UHS-M), respectively, equalling maximum resolution. The formula of the NEMA was used [16]:

$$ \mathrm{Uniformity}\ \left(\%\right)=100\times \frac{\operatorname{Max}\ \mathrm{count}-\operatorname{Min}\ \mathrm{count}}{\operatorname{Max}\ \mathrm{count}+\operatorname{Min}\ \mathrm{count}} $$

### Contrast-to-noise ratio

For the assessment of the contrast-to-noise ratio (CNR), the method firstly introduced by Walker et al. [17] was applied. Therefore, a template was created by drawing ROIs on a high-resolution computed tomography (CT) image of the two mini Derenzo phantoms 850.100 and 850.500. After setting the length to 6.0 mm with 0.9 times the diameter of the rods, the ROIs were placed on SPECT images with changing collimators as shown in Fig. 2. Circles in red and blue indicate hot and cold areas, respectively.

The contrast *C*_*d*_ and noise *N*_*d*_ were calculated as follows:

$$ {C}_d=\frac{\overline{R_d}-\overline{B_d}}{\overline{R_d}}\kern1em {N}_d=\frac{\sqrt{\sigma_{R_d}^2+{\sigma}_{B_d}^2}}{\overline{ROIs_d}} $$

The calculation was performed for each rod diameter *d*. $\overline{R_d}$ represents the mean value of all ROIs placed in the hot rods and $\overline{B_d}$ represents the mean value of all ROIs placed on cold areas in-between. For noise calculations,${\sigma}_{R_d}$and ${\sigma}_{B_d}$ are the standard deviations of hot *R*_*d*_ and cold *B*_*d*_ rods, respectively, while $\overline{ROIs_d}$ is the mean value of all ROIs for the investigated diameter *d*.

The contrast-to-noise ratio was defined as:

$$ {\mathrm{CNR}}_d=\frac{C_d}{N_d} $$

SPECT images were post-filtered by applying a 3D-Gaussian filter. FWHM was set to the diameter of each investigated rod section to enhance image quality and maximise the CNR. The above mentioned three different collimators XUHR-M, GP-M and UHS-M were investigated by using two activity concentrations of 32.8 ± 4.1 MBq/ml and 320.1 ± 6.5 MBq/ml, respectively. The acquisition time was set to 3 s, 30 s and 300 s time per bed position (TPB).

To illustrate the comparison of the individual count levels, the scans were divided into the activity concentrations that would have achieved the same results with a TPB of 300 s. Consequently, the descriptors used are ~ 0.3 MBq/ml, ~ 3 MBq/ml, ~ 30 MBq/ml and ~ 300 MBq/ml with a total scan duration of 45 min, since at least 9 BP were required to cover the examined volume completely.

Thus, the image quality achieved with the three different collimators for rods from 0.35 to 1.50 mm was compared for a wide range of count levels. In addition, the images of the different activity concentrations obtained were examined visually for the respective collimator- and count-dependant maximum resolution in order to be able to better assess and classify the CNR values.

### Comparison to U-SPECT^+^

A conventional SPECT system with three stationary detectors (U-SPECT^+^; MILabs), in the following referred to as “3DT-Scanner”, was used to compare the new 2DT-Scanner with two large stationary detectors to an established standard of reference in preclinical imaging of small rodents.

The measurements were performed analogously with the isotope ^99m^Tc in the same 850.100 phantom and the same GP-M collimator as in the 2DT-Scanner to compare imaging capabilities of these two systems.

For sensitivity assessment, the activity was 3.7 MBq as a point source. Acquisition parameters were kept as mentioned above with 1 BP and a total scan time of 5 min. For examining the spatial resolution, we used the 850.100 (Fig. 2) mini Derenzo phantom with an activity concentration of 296.4 MBq/ml, total acquisition time of 60 min, 12 BP and 300 s TPB. The uniformity was also calculated similarly to the examined 2DT-Scanner with the help of the 850.100 phantom without insert. Activity concentration was 298.6 MBq/ml with a filling volume of 1.5 ml. Acquisition time was set to 60 min (12 BP, 300 s TPB). For CNR analysis, the activity concentration was 298.1 ± 1.6 MBq/ml with acquisition times of 3 s, 30 s and 300 s TPB. These scans were divided into ~ 3 MBq/ml, ~ 30 MBq/ml and ~ 300 MBq/ml for 300 s TPB and total scan duration of 45 min.

All images acquired by the 3DT-Scanner were reconstructed using POSEM [12] with 32 subsets and 12 iterations, as this was the recommended reconstruction method with the same number of total updates of 384. Post-processing and performance parameter calculations were done in the same way as described for the 2DT-Scanner in the sections above.

## Results

### Sensitivity

The sensitivity measured with the XUHR-M collimator was 237 cps/MBq (0.024%), for the GP-M collimator 847 cps/MBq (0.085%) and for the UHS-M collimator 2054 cps/MBq (0.205%). Sensitivity for the established 3DT-Scanner was 1710 cps/MBq (0.171%) and so more than twice the sensitivity of the 2DT-Scanner system with one detector less and the same GP-M collimator.

### Spatial resolution

**Fig. 3:**
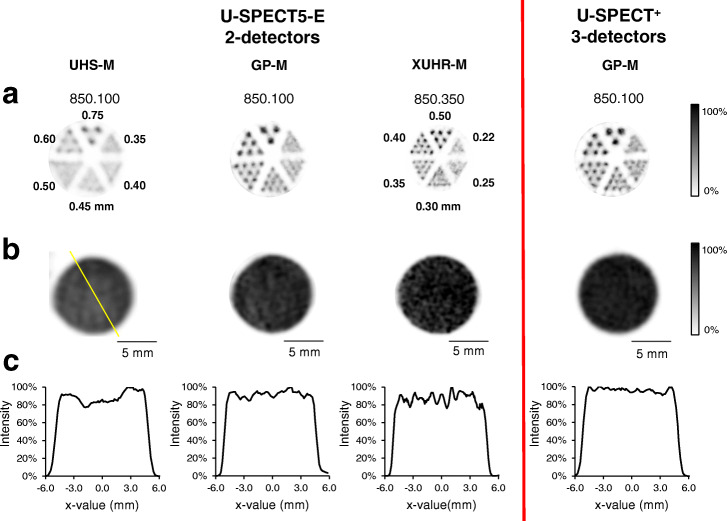
SPECT images for assessment of spatial resolution with respective hot-rod phantoms (**a**) and uniformity with homogenously filled phantoms (**b**). **c** Shows corresponding line profiles illustrated as yellow line in (**b**). Contrasting juxtaposition of the three investigated collimators UHS-M, GP-M and XUHR-M. Right side shows data acquired with the 3DT-Scanner. Smallest discriminable rod size was 0.75 mm for the UHS-M, 0.50 mm for the GP-M (2DT-Scanner and 3DT-Scanner), 0.35 mm for the XUHR-M. Uniformity records 23.0% (UHS-M), 31.2% (GP-M), 16.3% (GP-M, 3DT-Scanner) and 41.3% (XUHR-M). Representative images were chosen with slice thickness of 0.2 mm for UHS-M and GP-M and 0.1 mm for XUHR-M, respectively, for optimal visualization

For the investigated collimators, rod diameters of 0.35 mm for the XUHR-M, 0.50 mm for the GP-M and 0.75 mm for the UHS-M could be resolved. Despite the third detector, the 3DT-Scanner was not able to clearly resolve rods smaller than 0.50 mm with the same GP-M collimator. In Fig. 3a**,** hot-rod studies are shown to compare each collimator.

### Uniformity

By calculating the uniformity in accordance to the NEMA protocol, the results for the investigated collimators were 41.3% (XUHR-M), 31.2% (GP-M) and 23.0% (UHS-M). The 3DT-Scanner achieved a uniformity of 16.3% with the GP-M collimator and was therefore superior to all collimators examined in 2DT-Scanner. Reconstructed images and line profiles are shown in Fig. 3a, b for illustration.

### Contrast-to-noise ratio

**Fig. 4:**
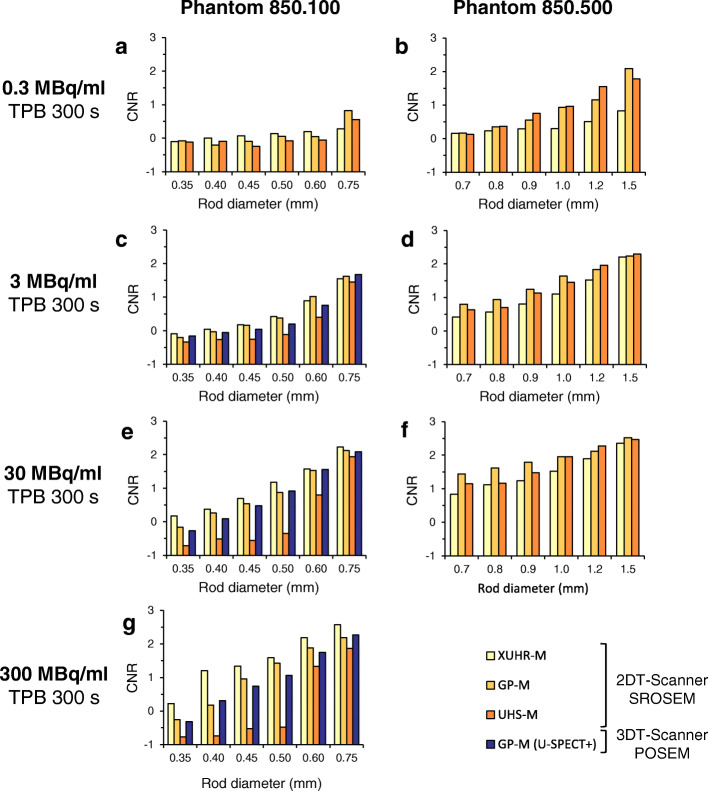
Contrast-to-noise ratio (CNR) of the investigated collimators with optimized values for each rod size by Gaussian post-filtering (full width half maximum = rod size). Bar charts compare the image quality of the collimators XUHR-M, GP-M and UHS-M for different activity levels in the mini Derenzo phantoms 850.500 and 850.100. In addition, the graphs in (**c**), (**e**) and (**g**) show how the 3DT-Scanner with the same GP-M collimator and a pixel-based ordered-subset expectation maximisation (POSEM) reconstruction algorithm compares to the new 2DT-Scanner and the similarity-regulated ordered-subset expectation maximisation (SROSEM) algorithm. Time per bed position (TPB) was 300 s with at least 9 bed positions (BP), corresponding to a total scan duration of 45 min

**Fig. 5:**
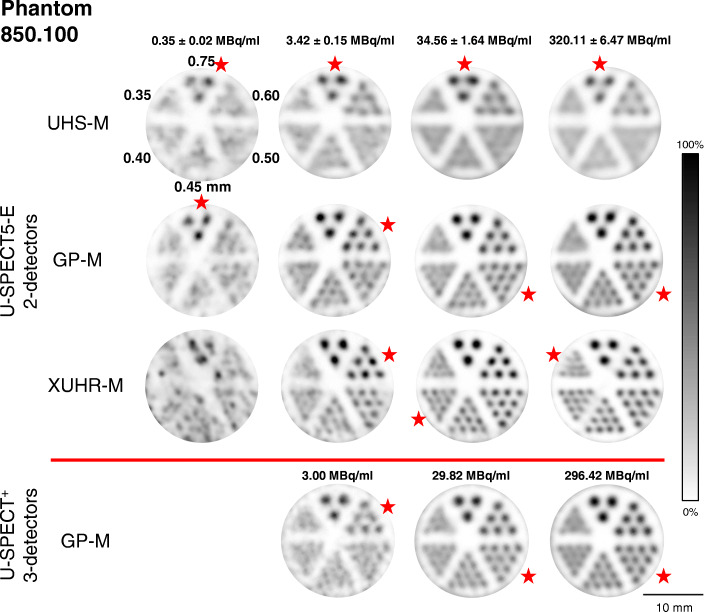
Reconstructed SPECT images of the mini Derenzo phantom 850.100 with various activity concentrations (mean ± standard deviation) of ^99m^Tc for a 300 s TPB scan (9 BP 2DT-Scanner, 12 BP 3DT-Scanner). Rod sections marked with the red star were assumed to be the smallest diameter resolved. All images are shown with 6.0 mm slice thickness and 0.35 mm full width half maximum Gaussian post-filter

**Fig. 6:**
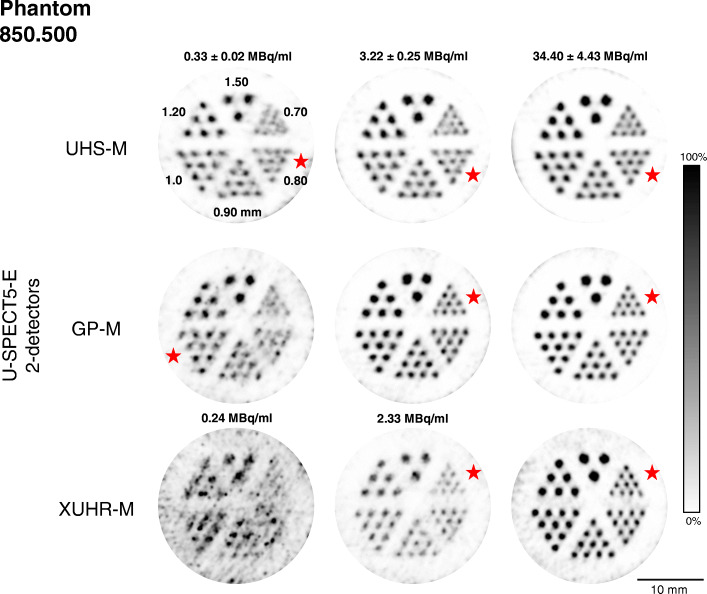
Reconstructed SPECT images of the mini Derenzo phantom 850.500 with various activity concentrations (mean ± standard deviation) of ^99m^Tc for a 300 s TPB scan (10 BP). Rod sections marked with the red star were assumed to be the smallest diameter resolved. All images are shown with 6.0 mm slice thickness and 0.35 mm full width half maximum Gaussian post-filter

Figure 4 shows the CNR values for four different count ranges, plotted against the rod diameter of the examined mini Derenzo hot-rod phantoms. The reconstructed images used for calculation are shown in Figs. 5 and 6 for illustration.

### UHS-M

The UHS-M showed its potential in the low-count setting with 0.3 MBq/ml activity concentration for 0.80 to 1.20 mm rod diameters with CNR ranging from 0.37 to 1.55. The maximum value was reached for 1.50 mm and is 1.78, which is still smaller than that of the GP-M with 2.09. Also, for the 3.0 MBq/ml study, it reached the highest CNR values for 1.20 and 1.50 mm with 1.96 and 2.29, respectively. Close to the assessed maximum resolution of 0.75 mm, the UHS-M achieved a usable image quality with a peak CNR of 1.94 for the 30 MBq/ml measurement. CNR values decreased again for the 300 MBq/ml measurement with the image quality for the high-count setting being reasonable. The overall peak CNR was achieved for the 30 MBq/ml measurement with the 1.50 mm rod size and was 2.47.

### GP-M

The general-purpose mouse collimator was superior for intermediate count range of 3.0 MBq/ml, especially in the range of 0.60 to 1.00 mm. For the rods of the 850.100 phantom, the CNR ranged from 0.76 for 0.60 mm to 1.62 for 0.75 mm. CNR in the 850.500 phantom for rod sizes of 0.70 to 1.00 mm were 0.80 to 1.64. For the 0.3 MBq/ml measurement, the GP-M was able to show strength for 0.75 mm with 0.82 and for 1.50 mm with 2.09. For the high-count setting with 30 MBq/ml, it still was able to achieve the best image quality for 0.70 to 1.00 mm and 1.50 mm. For the collimator’s maximum resolution of 0.50 mm, CNR values were 0.05 to 1.42, only resolved starting with the activity concentration of 30 MBq/ml. The overall peak CNR was 2.52 for 1.5 mm rods in the 30 MBq/ml measurement.

### XUHR-M

For the low-count study, the XUHR-M was not able to show any advantages in CNR. Even though Fig. 4 suggests a supposedly better image quality, the rods were not resolved as shown in Fig. 5. CNR was low and even partially negative ranging from − 0.10 for 0.35 mm to 0.83 for 1.5 mm. For the studies with 3.0 MBq/ml up to 300 MBq/ml, the XUHR-M showed a rod diameter range with superior CNR, being 0.35 mm with − 0.10 to 0.50 mm with 0.43 for 3.0 MBq/ml and 0.35 to 0.75 mm for 30 and 300 MBq/ml with 0.18 to 2.23 and 0.22 to 2.57, respectively. The visually assessed maximum resolution of 0.35 mm could only be achieved for the studies with 30 and 300 MBq/ml in which the corresponding CNR value was 0.18 and 0.22, respectively. Maximum CNR was calculated for 0.75 mm of the 300 MBq/ml study and was 2.57.

### GP-M: U-SPECT5 E-Class vs. U-SPECT^+^

Comparing the 2DT-Scanner with the 3DT-Scanner for the maximum resolution of 0.50 mm, CNRs were 0.38 and 0.20 for 3 MBq/ml, 0.87 and 0.92 for 30 MBq/ml and 1.42 and 1.06 for 300 MBq/ml, respectively. The total maximum CNR for the compared rod range from 0.35 to 0.75 mm of the 850.100 phantom was lower for the 2DT-Scanner with 2.19 than for the 3DT-Scanner with 2.27 for 0.75 mm with 300 MBq/ml. Overall, the values of the two systems only differed by an average of 3.6%. As shown in Fig. 5, the image quality was comparable.

## Discussion

We carried out a performance evaluation looking at sensitivity, spatial resolution, uniformity and contrast-to-noise ratio of a novel, ultra-high-resolution, small-animal SPECT with two large stationary detectors. Using three different multi-pinhole collimators for mouse imaging, we compared the scanner’s performance to a conventional, three-detector micro-SPECT system. The sensitivity of the new 2DT-Scanner was reasonably high, considering the reduced detection area by one third. Concerning uniformity, the UHS-M (23.0%) and GP-M (31.2%) collimators were clearly superior to the XUHR-M (41.3%) with the U-SPECT5-E. Nonetheless, the three-detector system with a homogeneity of 16.3% (GP-M) surpassed the two-detector system in that regard. We assume that this decrease in uniformity is due to a combination of lower sensitivity and the lack of corrective image information from the third bottom detector [18]. Even without the bottom detector, however, the two investigated collimators UHS-M and GP-M were able to achieve ultra-high spatial resolution in the submillimetre range for high-count as well as low-count settings of approximately 0.3 MBq/ml activity concentration. For the XUHR-M collimator, a concentration of around 3.0 MBq/ml was necessary to achieve a maximum resolution of smaller than 1.00 mm. The smallest discriminable rod size in our experiments was 0.75 mm (UHS-M, with 2DT-Scanner), 0.50 mm (GP-M, with both the 2DT-Scanner and 3DT-Scanner) and 0.35 mm (XUHR-M, with 2DT-Scanner), respectively. When comparing the 2DT-Scanner and 3DT-Scanner, maximum resolution remained unchanged, as this depends in particular on the design of the collimator and its pinhole size which was the same for both systems. The loss of sensitivity can be explained by the reduced total detector surface, as this is decisive for the maximum number of registered counts [19].

We decided to use visual analysis of hot-rod phantoms instead of line spread functions, as we focused on the tomographic resolvability of the activity-filled rods to allow better transfer to preclinical in vivo settings. CNR analysis was performed to evaluate the image quality of three collimators for different purposes of mouse imaging, as well as comparing the CNR to a conventional 3DT-Scanner system with three stationary detectors. It should be mentioned that due to the reduced number of rods with a diameter of 0.75 mm in the 850.100 phantom, the CNR for this section became larger than for 0.80 and 0.90 mm in the 850.500 phantom, which makes a direct comparison between them impossible. The UHS-M collimator provided the best image quality in the low-count setting of 0.3 MBq/ml, mainly because of its high sensitivity. A higher activity concentration of 3.0 MBq/ml was also associated with significantly better image quality. Despite the increasing CNR, it was found that by further increase of the count level, the advantages of the high sensitivity became less noticeable, because starting from about 3.0 MBq/ml, the GP-M collimator showed higher values for almost all rod sizes. At least an intermediate activity concentration of 3.0 MBq/ml was necessary to receive excellent contrast and noise for the GP-M. Low-count image quality was still reasonable for the GP-M with minor deficits compared to the UHS-M. Assessing the small rods of 0.35 to 0.60 mm, the image quality of the XUHR-M with 3.0 MBq/ml was, albeit not good, comparable to the other collimators. High activity concentrations of preferably at least 300 MBq/ml were essential for resolving small structures with beneficial contrast-to-noise ratio. Regarding the XUHR-M collimator, further experiments with even higher activity concentrations are necessary to fully exploit its potential. With the pinhole size of 0.25 mm, a resolution of about 0.25 mm should be achieved under ideal conditions, as already described [7]. In addition, a resolution of 0.15 mm in a mini Derenzo phantom was achieved with another collimator for tissue samples [20]. It has to be conceded, however, that high activity concentrations, long scan times or focus on a very small FOV might not be realistically transferable to in vivo settings.

Comparing the 2DT- to the 3DT-Scanner regarding the capabilities of the GP-M collimator, results suggest that both scanners have similar image quality performance for the examined rod diameters. Yet the 2DT-Scanner was able to reach slightly higher CNR values for the majority of investigated rod sizes despite the smaller detection area. As the CNR of the three-detector system deviates only 3.6% in average from the two-detector system, a clear advantage in image quality could not be determined. While this may be attributed to the novel system’s performance in the first place, it must also be taken into account that different reconstruction algorithms were used. Acquisition and reconstruction parameters were defined as comparable as possible, though. Based on our collimator comparison, we can support the manufacturer’s recommendation to use the GP-M collimator for general purposes, as it provides the best image quality in most cases. UHS-M could be advantageous especially for whole body images that do not require very high image quality. XUHR-M may be suitable for overnight scans of dead animals with high injection dose and high uptake values in the target organ.

The results for the 2DT-Scanner in our work are largely consistent with earlier studies on the performance of its established predecessor U-SPECT-II [10]. The tested scanner maintains the characteristic advantages and disadvantages of ultra-high-resolution multi-pinhole SPECT with stationary detectors compared to opponents with moving detectors [3, 21–23].

Although one detector was removed, the image quality of 2DT-Scanner was on par compared to an established three-detector set-up, especially under low-count conditions. Therefore, we expect the U-SPECT5 three-detector system to be even better due to higher sensitivity and uniformity. While the advantages of a third detector in terms of sensitivity could be compensated by increasing either the injection dose or the detection time by about 50%, it should be noted that injection volume, radioactive dose and scanning time are strictly limited in many places due to animal welfare regulations [24]. Therefore, focused scans could help to maintain high spatial resolution with reasonable image quality, e.g. by applying the method introduced by Branderhorst et al. [25]. If a very high in vivo resolution is required, it may be possible to achieve this by using a precisely defined scan volume and an application for defining such volumes has already been described [9]. Depending on the research question and the selected scan volume, important information may be lost during this procedure.

For the future perspective, multi-isotope studies would be of interest, as recently published for another SPECT scanner [26]. Also, when using several isotopes, an approach in the low-count range could be investigated, as it might be more realistic concerning in vivo performance.

Finally, we are looking forward to a standardised phantom and performance protocol for small animal SPECT, as well as it is established by NEMA for small animal PET [16].

In the future, it will be of interest how the measured performance of the system will be transferable to an in vivo mouse study and how the 2DT-Scanner performs with further established isotopes as well as in multi-isotope setting.

## Conclusion

The novel U-SPECT5-E system evaluated in this study provides first-rate spatial resolution and contrast-to-noise ratio with two stationary detectors comparable to the performance of an established three-detector scanner. Despite decreased sensitivity and signal uniformity in the absence of a third detector, the tested scanner’s image quality is suitable for preclinical SPECT imaging in small animals.

## Data Availability

The datasets used and analysed during the current study are available from the corresponding author on reasonable request.
